# Evaluating mental health education in ethnic minority universities in China: a CIPP model approach

**DOI:** 10.3389/fpsyg.2025.1679742

**Published:** 2025-11-10

**Authors:** Lu Zhang, Wenhui Wu

**Affiliations:** 1 School of Educational Science, Inner Mongolia Minzu University, Tongliao, Inner Mongolia, China; 2 Inner Mongolia Ethnic Education and Psychological Development Research Base, Tongliao, Inner Mongolia, China; 3 Inner Mongolia Student Bullying Prevention Research Center, Tongliao, Inner Mongolia, China; 4 Department of Student Affairs (Department of Armed Forces), Inner Mongolia University of Technology, Hohhot, Inner Mongolia, China

**Keywords:** mental health education, CIPP, ethnic minority students, higher education, program evaluation

## Abstract

**Introduction:**

The growing need for mental health education in ethnic minority universities in China has highlighted the importance of evaluating program effectiveness. This study aimed to develop and validate a CIPP-based scale designed to assess the quality and impact of mental health education programs in these institutions.

**Methods:**

A total of 1,635 students from 10 universities in Inner Mongolia participated in the study. The CIPP-based scale was tested for reliability and validity across four dimensions—context, input, process, and product—using quantitative analyses.

**Results:**

The results demonstrated strong structural validity and internal consistency of the scale. Significant group differences emerged in student evaluations by gender, academic year, discipline, and institution type, while prior counseling experience had no significant influence.

**Discussion:**

Although the program was broadly recognized for its effectiveness and instructional quality, challenges persisted in student engagement, resource continuity, and service utilization. The findings underscore the need for an integrated, inclusive, and culturally responsive educational framework to enhance mental health education for ethnic minority students.

## 1 Introduction

Globalization and digitalization amplify academic and public concern regarding university students’ mental health. As a high-risk population, college students often struggle with issues such as environmental adjustment and academic stress, making them particularly vulnerable to psychological difficulties (Zhang et al., 2024). Depression, anxiety, and OCD symptoms are highly prevalent among this group (Beiter et al., 2015; American College Health Association, 2019; Chen, 2025; Ning et al., 2024; Tuersun et al., 2025). The COVID-19 pandemic intensified mental health challenges among university students (Cao et al., 2020; Tang et al., 2020; Li et al., 2021; Roche et al., 2024), creating unprecedented complexities for higher education support systems. This crisis revealed a critical gap: rising demand for psychological services contrasted sharply with persistently low student utilization rates (Hunt and Eisenberg, 2010; Adams et al., 2022; Qiu et al., 2024), exposing institutional shortcomings in mental health provision.

Within university campuses, students are not only confronted with the challenges of adjusting to a new environment (Blakemore, 2019; Gao et al., 2024; Zhao B. et al., 2023), but also face increasing academic demands and a variety of stressors extending beyond the classroom. These include social, economic, and emotional pressures that can significantly impact their wellbeing (Suresh et al., 2021). Such stressors have been linked to learning difficulties and the exacerbation of mental health problems, anxiety, depression, poor sleep quality, and, in severe cases, suicidal ideation (Auerbach et al., 2016; Stone et al., 2015; Tang et al., 2018; Watkins et al., 2012; Li et al., 2025; Su et al., 2025). A large-scale survey involving 120,000 college students revealed that approximately 20% reported experiencing psychological distress (Li et al., 2008), highlighting the widespread nature of mental health challenges in this population (Chen et al., 2024; Han et al., 2025).

Demographic and contextual factors such as gender and geographic origin significantly influence mental health outcomes among university students in China (Liu et al., 2023; Wang S. et al., 2022). These challenges are particularly pronounced in ethnic minority regions, where cultural diversity, geographic isolation, and disparities in educational resources exacerbate barriers to effective psychological support. In such contexts, the delivery of mental health education is frequently hindered by limited faculty capacity, insufficient teaching materials, and cultural incongruities, all of which constrain the ability to meet students’ psychological needs.

Ethnic minority students account for approximately 9.63% of China’s university student population (Wang et al., 2025), with enrollment rates showing a consistent upward trend (Zhang et al., 2025). These students are predominantly from the western and border regions of the country. While most remain within their local communities during primary and secondary education, transitioning to university often entails relocation to distant institutions, which may introduce significant psychological stress. Studies have shown that relocation can intensify stress among ethnic minority students due to substantial changes in environment and lifestyle, increasing the risk of psychological distress (Gaw, 2000; Thomas and Choi, 2006; Ye, 2016; Zhao, 2023a; Zhou and Wang, 2023; Zhou, 2023b), and in more severe cases, contributing to depressive symptoms (Honglv et al., 2023; Li, 2007). Students from remote areas may also exhibit lower cognitive and psychological assessment scores (Zhang et al., 2025), further heightening their vulnerability to mental health issues.

The body of research focusing on ethnic minority students’ mental health is growing, with current studies centering on three main domains: (a) the assessment of psychological wellbeing, (b) the identification of contributing psychological risk and protective factors, and (c) the evaluation of intervention effectiveness within this population.

Ethnic minority college students face prominent stressors in cultural adaptation—such as language barriers, religious differences, and acculturative anxiety—which have been consistently linked to psychological distress in recent studies (Mu and Jiang, 2018; Yu and Guo, 2024; Zhou, 2023b). They advocated for institutional strategies aimed at fostering inclusive campus climates and promoting intercultural communication. In a complementary study, Yin (2021) found that graduate students from economically disadvantaged and minority backgrounds in central and western China experienced disproportionately high mental health burdens. This regional disparity aligns with findings by Wang S. et al. (2022), who documented higher incidences of mental health concerns among students in Inner Mongolia.

Sun et al. (2023) observed elevated levels of social anxiety among Tibetan adolescents residing in high-altitude regions, which were strongly associated with sleep deprivation. Meanwhile, Zhang et al. (2025) reported generally positive mental health among ethnic minority university students but noted shortcomings in cognitive flexibility and adaptive functioning. These findings stand in contrast to Yin’s (2021) emphasis on academic stress and financial pressures as the primary determinants of mental health challenges in graduate students from central and western regions Xin and Liu’s (2019). Cross-temporal meta-analysis identified substantial improvements in the mental health of ethnic minority college students over time, with some studies even reporting lower levels of mental distress compared to Han students (Wang Z. et al., 2022). Collectively, these varied findings underscore the multifaceted nature of psychological outcomes among ethnic minority groups, shaped by environmental, socioeconomic, and developmental variables.

In terms of program evaluation Jiang and Ren (2011), adapted the CIPP model—a decision-oriented framework originally developed by Stufflebeam in the late 1960s—into a tool for assessing school-based mental health education (Stufflebeam, 1968; Stufflebeam, 2003). The model comprises four dimensions: Context, Input, Process, and Product and emphasizes a comprehensive approach that spans program planning, implementation, and refinement. Recent evaluations in areas such as teacher education, medical training, and curriculum reform show that CIPP remains a practical tool for guiding program refinement and evidence-based decision making. Although the model has been widely applied across education and health settings, its use specifically in school-based mental health education is still limited, which highlights the contribution of the present study (Stufflebeam, 1983; Razack et al., 2007; Chanthalangsy et al., 2024; Gerayllo et al., 2025; Dizon, 2023; Liu et al., 2023; Sankaran and Saad, 2022).

Together, these studies offer both theoretical insights and methodological guidance for enhancing mental health education in higher education. Although their findings vary, they collectively affirm the essential role of mental health education in supporting student wellbeing. Several scholars have proposed actionable recommendations, including improvements in program design and faculty training (Ma and Feng, 2022; Xi and Zhang, 2024), providing practical pathways for universities. Nevertheless, the current literature remains disproportionately focused on Han students, with limited attention to the psychological development of ethnic minority groups. This research gap may hinder efforts to support the psychological and sociocultural adjustment of minority students within increasingly diverse academic environments.

Compared with previous studies evaluating mental health education in Chinese universities, the present study has several distinctive features. First, most prior research has focused on general student populations or specific courses, whereas our study specifically examines ethnic minority universities, which have unique cultural and institutional contexts (Han et al., 2025; Lin et al., 2025). Second, we adopted the CIPP model to assess the background, input, process, and product of mental health education, moving beyond one-dimensional or descriptive evaluations (Shang et al., 2022). While CIPP has been used in recent studies of educational and health programs, it has seldom been applied to school-based mental health, highlighting the distinct contribution of this work (Sankaran and Saad, 2022). Third, with a large sample of 1,635 students across 10 universities, our study offers higher statistical power and more generalizable insights (Cai et al., 2025; Fan et al., 2025). Overall, this study contributes to the literature by combining a structured evaluation model with culturally contextualized data, highlighting areas for improvement and providing guidance for tailored mental health interventions in ethnic minority higher education settings.

To address this gap, we employ the CIPP model to comprehensively evaluate mental health education in ethnic minority regions. Focusing on the College Students’ Mental Health course, we establish a systematic framework to: (a) identify pedagogical and curricular challenges, (b) propose evidence-based improvements, and (c) ultimately enhance psychological wellbeing and holistic development among minority students.

## 2 Development of an evaluation framework based on the CIPP model

### 2.1 Applying the CIPP model to assess the college students’ mental health course

To advance higher education in ethnic minority regions, China’s Ministry of Education prioritizes dual objectives: enhancing educational quality and advancing equity. The application of the CIPP model offers a scientifically grounded methodological approach to assess and enhance mental health education within universities serving these regions.

Context evaluation clarifies the sociocultural background and specific needs of students regarding mental health education. Input evaluation focuses on the allocation and efficiency of educational resources. Process evaluation monitors the implementation of instructional activities, while product evaluation assesses the actual outcomes of the educational programs. The specific evaluation content and indicator system employed in this study are outlined as follows.

### 2.2 Context dimension

The evaluation of the context dimension aims to understand the prevalence of mental health issues faced by university students on campus, as well as students’ prior knowledge and awareness of mental health concepts before taking the “College Students’ Mental Health Education” course. Additionally, it assesses students’ recognition of the importance of mental health education within the broader educational objectives.

Indicators within this dimension help analyze the demand and urgency for mental health education, providing essential data to inform subsequent goal setting. Specifically, Item 1: The prevalence of mental health problems gauges the extent to which students experience psychological difficulties, reflecting the necessity for mental health education. Item 2: Students’ understanding of mental health concepts evaluates their baseline knowledge prior to course participation, supporting the needs analysis. Item 3: gauges student recognition of mental health education’s role within broader educational objectives.

### 2.3 Input dimension

The input dimension focuses on the resources and support invested by the university in mental health education. This includes the extent to which mental health educators, counselors, and administrative staff attend to students’ psychological wellbeing, the availability and allocation of relevant facilities and resources, as well as the professional qualifications of personnel involved.

Assessment of this dimension reflects whether the institution provides adequate support in resource allocation to ensure the effective delivery of mental health education. Specifically, the attention given by mental health teachers, counselors, and administrative staff to students’ mental health concerns (Items 4–6) gauges the degree of staff engagement and prioritization of mental health. Item 12: The adequacy of mental health education resources evaluates the availability and distribution of facilities and materials. Finally, the quantity and professional competence of mental health personnel (Items 13–14) are examined to assess the quality of human resource investment.

### 2.4 Process dimension

The process dimension focuses on the implementation of mental health education courses and student engagement levels. This dimension evaluates: (a) the scientific rigor and practical relevance of the curriculum; (b) instructor teaching competence; (c) the adaptability of course content; (d) the interactivity of instructional methods; and (e) student participation and enthusiasm in related activities. Furthermore, it investigates the establishment of institutional mechanisms to ensure comprehensive stakeholder involvement and continuous evaluation throughout course delivery. Collectively, the indicators within this dimension gauge both the effectiveness and engagement inherent in the mental health education process. Specifically: Item 15: Assesses how well the course content aligns with established mental health education standards and reflects overall content quality. Items 22–23: Evaluate the implementation and effectiveness of university systems for tracking progress and gathering feedback during the mental health education process.

### 2.5 Product dimension

The product dimension focuses on the actual outcomes of the mental health education program, including students’ overall satisfaction with the course and related activities, the extent to which educational objectives are achieved, improvements in students’ mental health status, and changes in their attitudes toward psychological wellbeing. This dimension also examines the integration of mental health education into students’ daily academic and personal lives, its positive impact during critical periods, and the long-term effects it fosters.

Evaluation within this dimension involves measuring overall course satisfaction (Items 7 and 25) to gauge students’ general contentment with the mental health curriculum and associated programs. The penetration of mental health education into daily learning and its role during key transitional periods (Items 28–29) are assessed to understand its practical influence. The durability of its impact (Item 30) evaluates the sustained benefits of the program on students’ physical and psychological health.

## 3 Empirical study design and implementation

The participants in this study consisted of 1,635 undergraduate students from the 2023 and 2024 cohorts across 10 higher education institutions in the Inner Mongolia region. Ethical approval was obtained from the Institutional Review Board of (Inner Mongolia Minzu University), and all participants provided informed consent prior to completing the questionnaire. Data were collected through online surveys distributed in classrooms by mental health educators at the participating institutions. These educators are familiar with the characteristics and needs of minority students, enabling effective communication and enhancing both the validity and representativeness of the responses.

To ensure a representative sample, this study employed a stratified random sampling approach, balancing across institution type (undergraduate vs. vocational colleges), grade level, and academic discipline. This method enhances the precision of estimates by ensuring that all relevant subgroups are adequately represented, thereby improving the generalizability of the findings (Hu et al., 2023; Tao et al., 2025). The sample included students from diverse majors and backgrounds to capture a comprehensive profile of the student population. However, it should be noted that the sample exhibited a gender imbalance, with females accounting for approximately 73.8% of participants. This imbalance may affect the generalizability of the findings and was considered in subsequent analyses.

In addition, confirmatory factor analysis (CFA) was conducted to test the hypothesized factor structure and to further evaluate the structural validity of the scale, building on the results of the exploratory analysis. CFA has been widely applied in recent studies to validate instruments in education and mental health contexts (Liu et al., 2023; Zhao S. et al., 2023). Descriptive statistics of the participants are shown in Table 1, and all tables and figures were revised to ensure clarity and consistency with the analyses reported in the text.

**Table 1:** Basic information composition of the survey subjects.

Demographic distribution		Number	Proportion
Gender	Male	428	26.18%
	Female	1,207	73.82%
Grade	Grade 2023	556	34.62%
	Grade 2024	1,079	65.99%
Major	Liberal arts student	750	45.87%
	Science student	885	54.13%
Institution category	University	762	46.61%
	Academy	873	53.39%
Experience in psychological counseling	Yes	272	16.64%
	No	1,363	83.36%
Total		1,635	

## 4 Data analysis

### 4.1 Scale validation

We assessed the reliability and validity of the CIPP-based mental health education scale through the following analyses:

#### 4.1.1 Exploratory factor analysis (EFA)

Initial factor analysis yielded a Kaiser-Meyer-Olkin (KMO) index of 0.854—substantially exceeding the 0.60 benchmark—confirming robust variable inter correlations suitable for dimension reduction. Bartlett’s sphericity test reached significance (*χ*^2^ = 8871.659, *df* = 6, *p* < 0.001), rejecting the null hypothesis of variable independence.

Principal component analysis with Varimax rotation extracted four factors (eigenvalues > 1), corresponding to the CIPP framework’s domains: Context, Input, Process, and Product. These accounted for 78.874% of cumulative variance, demonstrating strong structural validity and theoretical alignment.

#### 4.1.2 Reliability analysis

Internal consistency reliability was assessed using Cronbach’s alpha coefficients. Results showed that the overall scale achieved a Cronbach’s alpha of 0.983, indicating excellent internal consistency. The Cronbach’s alpha values for each subscale were:

- Context Evaluation: 0.917
- Input Evaluation: 0.951
- Process Evaluation: 0.923
- Product Evaluation: 0.968

All subscales exceeded the commonly accepted threshold of 0.70, demonstrating high reliability and consistent internal coherence across dimensions.

#### 4.1.3 Confirmatory factor analysis (CFA)

To further evaluate the structural validity of the scale, a confirmatory factor analysis (CFA) was conducted following the exploratory factor analysis and reliability assessment. The initial CFA indicated that the model did not fully meet recommended fit standards, with SRMR (Standardized Root Mean Square Residual) and RMSEA (Root Mean Square Error of Approximation) exceeding ideal thresholds. Examination of standardized residuals and modification indices revealed that the first item in the Context dimension (context1) showed large residual covariance’s with multiple items within the same factor and a relatively low factor loading, suggesting that it did not adequately represent the intended construct in this sample. Additionally, residual correlations between items e5 and e6, and between e15 and e16, were theoretically justifiable due to their similar content.

To improve model fit, the first item in the Context dimension (context1) was removed. This adjustment alone resulted in modest improvement (CFI = 0.930, TLI = 0.923, RMSEA = 0.087, SRMR = 0.0274, *χ*^2^*/df* = 13.481). Subsequently, two pairs of theoretically justified residual correlations (e5 ↔ e6 and e15 ↔ e16) were allowed, which further enhanced model fit. The final revised model demonstrated substantially improved indices (CFI = 0.940, TLI = 0.934, RMSEA = 0.081, SRMR = 0.0259, *χ*^2^*/df* = 11.670). While *χ*^2^*/df* remained elevated due to the large sample size, both incremental and absolute fit indices indicated that the final model provided a more accurate representation of the latent structure. These modifications were guided by theoretical considerations and empirical evidence, ensuring that the conceptual integrity of the constructs was preserved.

The results confirm that the refined scale exhibits satisfactory construct validity and supports its use in subsequent analyses, including group differences across gender, grade, major, institution type, and psychological counseling experience.

### 4.2 Group differences analysis

To analyze group differences in perceived efficacy of CIPP-based university mental health education, this study conducted systematic comparisons of scores on the four CIPP dimensions (Context, Input, Process, Product) and all secondary indicators. Grouping variables included gender, grade level, major, institution type (undergraduate universities vs. vocational colleges), and prior experience with psychological counseling.

#### 4.2.1 Gender differences

Female students demonstrated significantly higher context evaluation than males on Context Evaluation (*t* = 3.701, *df* = 1,633, *p* < 0.001), Input Evaluation (*t* = 3.292, *df* = 1,633, *p* = 0.001), and Process Evaluation (*t* = 2.736, *df* = 1,633, *p* = 0.006). However, gender differences in Product Evaluation were not statistically significant (*t* = 1.796, *df* = 1,633, *p* = 0.073), suggesting that females generally hold more positive evaluations across most stages of mental health education effectiveness.

#### 4.2.2 Grade differences

Significant differences were observed between 2023 and 2024 cohorts on all four dimensions (*p* < 0.001). Specifically, freshmen in the 2024 cohort reported higher average scores than sophomores in 2023 on Context (*t* = 3.725, *df* = 1,633, *p* < 0.001), Input (*t* = 5.387, *df* = 1,633, *p* < 0.001), Process (*t* = 5.390, *df* = 1,633, *p* < 0.001), and Product Evaluations (*t* = 4.563, *df* = 1,633, *p* < 0.001). These results indicate that first-year students tend to perceive the effectiveness of university mental health education more positively.

#### 4.2.3 Major differences

Science majors outperformed arts counterparts across all dimensions (*p* < 0.001). Specifically, science students’ mean scores surpassed those of arts students on Context (*t* = 4.155, *df* = 1,633, *p* < 0.001), Input (*t* = 4.213, *df* = 1,633, *p* < 0.001), Process (*t* = 4.307, *df* = 1,633, *p* < 0.001), and Product Evaluations (*t* = 4.122, *df* = 1,633, *p* < 0.001). This suggests a generally more favorable perception of mental health education among science majors.

#### 4.2.4 Institution type differences (undergraduate vs. vocational colleges)

Comparisons revealed that students at undergraduate institutions scored significantly higher than those at vocational colleges across all dimensions (*p* < 0.001):

- Context Evaluation: *t* = 6.954, *df* = 1,633, *p* < 0.001
- Input Evaluation: *t* = 7.713, *df* = 1,633, *p* < 0.001
- Process Evaluation: *t* = 8.078, *df* = 1,633, *p* < 0.001
- Product Evaluation: *t* = 7.051, *df* = 1,633, *p* < 0.001

These findings indicate that undergraduate institutions are perceived to deliver superior mental health education in terms of resource investment, implementation processes, and outcomes.

#### 4.2.5 Psychological counseling experience differences

Contrary to other grouping variables, no significant differences emerged between students with or without prior psychological counseling experience across any dimension (*p* > 0.05):

- Context Evaluation: *t* = 0.704, *df* = 1,633, *p* = 0.533
- Input Evaluation: *t* = −0.023, *df* = 1,633, *p* = 0.982
- Process Evaluation: *t* = 0.315, *df* = 1,633, *p* = 0.753
- Product Evaluation: *t* = 1.597, *df* = 1,633, *p* = 0.111

This suggests that students’ personal counseling history does not significantly affect their overall perception of the university’s mental health education system, encompassing all CIPP dimensions. Regardless of counseling experience, students’ evaluations of institutional mental health education tend to be consistent.

Detailed statistical results for these group comparisons on the secondary indicators are presented in Table 2.

**Table 2:** Analysis of differences in questionnaire indicators.

Process	Tier-1 indicator	Tier-2 indicator	Gender	Grade	Major	Institution category	Counseling experience
			*t*	*t*	*t*	*t*	*t*
Context	1. Educational needs and objectives	1.1. Prevalence of issues	−4.33^***^	−0.478	0.32	2.95^**^	−0.90
		1.2. Student awareness of mental health issues	−3.56^***^	1.107	−3.84^***^	3.45^***^	1.00
		1.3. Staff awareness of mental health issues	−2.35^**^	3.641^***^	−4.17^***^	6.59^***^	0.61
		1.4. Clarity of mental health education objectives	−3.10^**^	4.490^***^	−3.65^***^	6.35^***^	0.85
		1.5. Alignment of objectives with student needs	−2.79^**^	4.474^***^	−3.57^***^	6.99^***^	0.55
Input	2. Resources and support	2.1. Emphasis placed on mental health education	−3.48^***^	4.619^***^	−3.40^***^	6.57^***^	0.26
		2.2. Budget adequacy for mental health education	−3.12^***^	5.237^***^	−4.71^***^	6.89^***^	−0.43
		2.3. Number of professional mental health educators	−2.36^**^	4.387^***^	−4.02^***^	7.60^***^	0.86
		2.4. Professional competence of mental health educators	−3.41^***^	5.873^***^	−3.56^***^	7.71^***^	−0.79
Process	3. Implementation and execution	3.1. Scientific rigor and practicality of curriculum content	−3.61^***^	5.189^***^	−3.20^***^	6.79^***^	−0.31
		3.2. Teacher instructional competence	−3.86^***^	5.078^***^	−3.79^***^	7.25^***^	−1.19
		3.3. Adaptability of curriculum content	−2.81^**^	4.471^***^	−3.85^***^	6.31^***^	1.12
		3.4. Diversity and interactivity of teaching methods	−2.53^**^	5.485^***^	−4.20^***^	8.75^***^	0.04
		3.5. Student participation rate	−1.58	4.731^***^	−4.59^***^	7.77^***^	1.73
		3.6. Holistic education approach	−2.22^*^	4.156^***^	−3.33^***^	6.32^***^	−1.11
		3.7. Continuous education process	−2.42^*^	5.278^***^	−4.06^***^	7.50^***^	0.09
Product	4. Effects and outcomes	4.1. Feedback and satisfaction with mental health activities	−1.62	5.220^***^	−3.80^***^	7.16^***^	1.13
		4.2. Changes in mental health knowledge and attitudes	−1.30	4.140^***^	−4.18^***^	5.96^***^	2.29
		4.3. Achievement of educational objectives	−2.05^*^	4.201^***^	−3.85^***^	6.81^***^	1.34
		4.4. Long-term effects of educational interventions	−1.96	4.165^***^	−3.54^***^	7.40^***^	1.14

^*^p < 0.05; ^**^p < 0.01; ^***^p < 0.001.

## 5 Discussion

The CIPP-based scale developed in this study to assess the effectiveness of university mental health education clearly reflects the four theoretically grounded dimensions—Context, Input, Process, and Product—and demonstrates strong internal consistency reliability across the overall scale and each sub-dimension. These findings establish a solid methodological foundation for subsequent analyses of mental health education outcomes in universities located in ethnic minority regions.

Furthermore, the study revealed significant differences in students’ evaluations of mental health education effectiveness based on gender, grade level, major and institution type, whereas students’ individual experience with psychological counseling did not significantly influence their perceptions. These patterns of difference offer valuable empirical evidence for deepening the understanding of factors affecting educational effectiveness and for designing targeted strategies to optimize mental health education.

The following discussion will focus on interpreting the survey results and identified issues within the framework of the four CIPP dimensions.

### 5.1 Context evaluation

The context evaluation revealed a structural mismatch between the current top-down design of mental health education and the actual needs of students. While 73.08% of students affirmed the institution’s commitment to mental health initiatives, and 74.56% acknowledged the scientific and practical value of the course content, a notable 83.36% reported never having used psychological counseling services (Eisenberg et al., 2007; Hunt and Eisenberg, 2010; Cyr et al., 2010; Gulliver et al., 2010; Macaskill, 2012; Ning et al., 2024; Qiu et al., 2024).

Despite the relatively low rate of service utilization, female students rated the contextual fit of the course significantly higher than male students (Context dimension: *t* = 3.701, *p* < 0.001), suggesting that women may experience greater subjective engagement and a stronger sense of relevance in mental health education.

Moreover, the study found that students at undergraduate universities expressed significantly higher recognition of contextual adequacy compared to those at vocational colleges (*t* = 6.954, *p* < 0.001). This finding echoes earlier research showing that ethnic minority students generally demonstrate good psychological profiles and high scores in personality traits, yet exhibit lower levels of cognitive quality and adaptability (Zhang et al., 2025). The differences observed here may reflect broader regional disparities in educational access and quality, as many ethnic minority students are concentrated in western and border areas of China. These results highlight the ongoing need to balance and optimize resource allocation to promote equitable access to mental health education.

### 5.2 Input analysis

The input analysis reveals a notable discontinuity in resource allocation across academic years. While faculty expertise is highly regarded—with 75.96% of students rating instructors’ professional competence positively—resource distribution appears markedly imbalanced. The sample of 2024 freshmen accounts for 65.99% of respondents, highlighting a structural bias that is reflected in the grade-level differences: first-year students provide the highest evaluations of resource input, and they also demonstrate more favorable ratings across the other three dimensions. This pattern underscores the importance of the freshman year as a critical period for implementing mental health education (Liu et al., 2023; Luo and Mohammed, 2023).

In contrast, evaluations from the 2023 cohort (upperclassmen) are significantly lower than those from freshmen, suggesting that psychological difficulties tend to emerge progressively as students advance through university (Roche et al., 2024). Moreover, less than 40% of students maintain sustained and active participation in mental health activities, reflecting an inadequacy in supportive mechanisms to effectively engage students over time.

### 5.3 Process evaluation

The process evaluation highlights a critical challenge in the implementation of mental health education: a disconnect between innovative teaching methods and deep student engagement. While 72.66% of students acknowledged the diversity of instructional approaches, sustained and meaningful participation remains low, with less than 40% of students actively involved over time.

Group comparison analyses revealed that female students scored significantly higher than males on process evaluation, a finding consistent with previous research suggesting that females’ greater emotional expressiveness and more frequent use of social support resources contribute to higher levels of perceived relevance and satisfaction with educational interventions (Jiang and Sun, 2024; Wang et al., 2024). Disciplinary differences were also evident, with science students consistently reporting more favorable evaluations than their peers in the humanities. This may reflect differences in cognitive style and learning adaptability: science students often exhibit stronger self-regulation and problem-focused coping, which align more closely with the structure and pedagogical design of current mental health curricula (Jiang and Sun, 2024; Wang et al., 2024). Institutional type emerged as another significant factor. Students from undergraduate universities rated the process dimension notably higher than those from vocational colleges (Process Evaluation: *t* = 8.078, *p* < 0.001), suggesting that disparities in resource availability, instructional quality, and institutional culture may influence perceived effectiveness. However, as the grade level increases, the sense of experience in the mental health education process significantly decreases, and the enthusiasm of senior students is not high (*t* = 5.390, *p* < 0.001).

While 70.52% of students expressed satisfaction with the current follow-up and monitoring mechanisms, the persistent gap between this recognition and the 83.36% non-utilization rate of counseling services points to a disconnect between perceived support structures and actual student behavior. The relatively positive evaluations observed in this study may partly reflect the psychological resilience of the surveyed population, rather than a direct outcome of the educational process itself.

### 5.4 Product evaluation and feedback

The product evaluation confirms the core value of the mental health education program, yet a sustainable, long-term support system has yet to be established. A substantial majority of students (95.41%) acknowledged tangible improvements in their mental health following the course, with 70.64% reporting that these positive effects were enduring, thereby validating the immediate effectiveness of the educational intervention.

Group analyses revealed that female students perceived the outcomes significantly more positively than males (Product Evaluation: *t* = 1.796, *p* = 0.073). Additionally, students at undergraduate universities rated the program’s effectiveness markedly higher than those at vocational colleges (*t* = 7.051, *p* < 0.001). This discrepancy may be attributed to vocational college students’ relatively weaker cultural foundation, lower academic motivation, and greater psychological challenges (Xi and Zhang, 2024).

Notably, while 73.08% of students recognized the institution’s emphasis on mental health, only 16.64% actually engaged in counseling services (reflecting the inverse of the 83.36% who reported never seeking such services). This gap highlights a critical bottleneck in translating educational effectiveness into a supportive and sustainable mental health ecosystem (Gulliver et al., 2010; Hunt and Eisenberg, 2010; Macaskill, 2012; Quinn et al., 2009; Adams et al., 2022; Ning et al., 2024; Qiu et al., 2024).

## 6 Limitations and future research directions

Several limitations of this study should be acknowledged. First, one item (context1) performed poorly, emphasizing the need for careful item evaluation and cultural adaptation when applying the instrument in ethnic minority populations. Second, the sample was drawn from a limited geographic region and exhibited a gender imbalance, with females comprising nearly three-quarters of participants, which may affect the generalizability of the findings. Consequently, the levels of psychological distress and service demand observed here may reflect, at least in part, the predominance of female participants. Caution is therefore required when extending these results to male students, whose psychological experiences may follow different patterns. Addressing this limitation will require future studies to employ strategies such as quota sampling or inter-university collaboration, so that a more balanced gender distribution can be achieved and potential gender-specific differences more accurately examined. Third, the study relied exclusively on self-reported data, which may introduce response bias. Although the CIPP model provides a structured framework for evaluating mental health education, it may not fully capture all relevant influencing factors. While CFA adjustments improved model fit, the large sample size contributed to a relatively high *χ*^2^*/df*, which should be interpreted with caution. Future research could extend the scale validation to additional regions and ethnic groups, refine or remove problematic items, employ a combination of quantitative and qualitative methods, and integrate multiple data sources to enhance construct validity and applicability.

## 7 Conclusions and educational implications

Drawing on 1,635 valid questionnaires from 10-plus universities in Chinese ethnic minority regions, this study comprehensively assesses mental health education across four domains: course awareness, resource allocation, instructional effectiveness, and student engagement. The findings indicate that 95.41% of students reported significant improvements in their mental health, confirming the core educational value of the program. Furthermore, 74.56% recognized the scientific rigor of the course content, and 75.96% gave high ratings to faculty competence, reflecting the initial establishment of a foundational support system.

Most students perceive mental health issues on campus as prevalent and acknowledge the course’s positive impact on psychological wellbeing. However, some institutions demonstrate room for improvement in resource distribution, inclusive participation, and follow-up evaluation mechanisms.

The report aims to provide empirical support for mental health education in Inner Mongolia’s higher education institutions, and recommends establishing coordinated mechanisms between undergraduate and vocational programs, with a focus on bridging the support gap for upper-year students. It suggests developing culturally contextualized activities tailored to ethnic minority backgrounds to enhance engagement in vocational colleges and building an integrated “course-service-ecosystem” model. To strengthen cultural responsiveness, mental health education in minority-region universities should be embedded into locally meaningful practices. Instead of presenting psychological concepts as abstract knowledge, teaching and practical activities can incorporate elements of regional culture. For instance, introducing dairy-production practices in classroom demonstrations can provide a concrete context for discussing stress management, while traditional Mongolian dance can be reframed as a collective exercise for emotional regulation and balance. Embedding such cultural resources into teaching not only creates a stronger sense of recognition and belonging among students but also helps transform high participation rates into genuine psychological gains. Furthermore, applying digital visualization tools to track students’ developmental trajectories allows educators to adjust interventions in a culturally sensitive manner, thereby improving both the adaptability and effectiveness of the curriculum. This model would embed culturally relevant psychological modules to address the identified 14.3% gap in educational goals, design gender-sensitive outreach channels to activate the 83.36% of students not currently engaged, and strengthen continuity of effectiveness across academic stages.

## Data Availability

The raw data supporting the conclusions of this article will be made available by the authors without undue reservation.
